# Interventions to Reduce COVID-19 Vaccine Hesitancy among Black and African American Individuals in the United States: A Systematic Literature Review

**DOI:** 10.3390/vaccines12090959

**Published:** 2024-08-26

**Authors:** Evelyn Masterson, Emma Anderson, Elena Savoia

**Affiliations:** Emergency Preparedness Research Evaluation and Practice Program, Division of Policy Translation and Leadership Development, Harvard T.H. Chan School of Public Health, 677 Huntington Avenue, Boston, MA 02115, USA

**Keywords:** interventions, vaccine hesitancy, COVID-19, Black, African American

## Abstract

COVID-19 vaccine hesitancy had major implications for racial health equity at the beginning of the vaccination campaign in the U.S. Interventions to reduce vaccine hesitancy among Black and African American individuals partially helped to reduce vaccine hesitancy in specific communities. This article describes findings on interventions to reduce COVID-19 vaccine hesitancy among Black and African American individuals from a literature review we conducted. We found 12 studies that described communication, partnerships, and distribution interventions. Regarding communication, examples include a webinar hosted by an academic-community partnership team, information sessions, social media campaigns, educational materials, and virtual town halls. Effective partnerships identified through this literature review were a statewide alliance and one between an academic institution and faith and community leaders. Distribution interventions identified through the literature review were the deployment of multiple tactics to increase COVID-19 vaccine uptake (virtual town halls, a confidential employee hotline, department huddles, written educational material, and accessible vaccination stations) and offering to administer the COVID-19 vaccine during medical appointments. The results of this review show that implementing interventions directed at specific minority groups improves COVID-19 vaccine acceptance without undermining overall vaccine distribution or uptake.

## 1. Introduction

As Finucane M.L. et al., state in their 2021 report on the assessment of racial equity in disaster preparation, response, and recovery, “Equity is a multidimensional construct typically characterized by fairness and a lack of self-interest [1,2]. Unlike equality, where the same treatment is given to all regardless of personal advantage or disadvantage (Sen 1992), equity allows for unequal distribution of benefits and costs for the sake of net social gain (McDermott et al. 2013)” [3,4,5]. Equity is always essential to strive toward, especially in preparation of or during a public health emergency when it becomes critical to align preparedness efforts and distribution of resources to achieve the greatest impact in reducing health disparities, particularly those that research has shown to be strongly linked to the demand for health care and preventive services, such as vaccinations. However, great care should be taken to ensure that efforts are sensitive and truly beneficial to the populations they reach. For example, the federal government’s attempt at improving racial health equity by prioritizing Black and African American individuals when rolling out the COVID-19 vaccine can easily be misinterpreted as using this population as “guinea pigs” for a new vaccine when the government has a history of exploiting Black and African American individuals to test treatments (or a lack thereof) [6], with the USPHS Untreated Syphilis Study at Tuskegee often cited as directly or indirectly influencing COVID-19 vaccine hesitancy among Black and African American individuals [7].

What is required to achieve equity is not static. The needs of a group, a subset of a group, or an individual are ever-changing in the face of a public health emergency because no sociodemographic group is a monolith at any level—national, state, or local. Within neighborhoods, there are trends that can be useful to policymakers, agencies, and researchers alike, but each individual person has unique strengths and needs that allow them to adapt more easily to an emergency or require additional assistance. Level of risk is determined by the interaction of hazard(s), vulnerability, and risk of exposure [8]; therefore, levels of risk differ both within and across geographic areas and segments of the population. Because of their close, often daily interactions with the public, local agencies play a critical role in determining who may be particularly vulnerable to the consequences of a large-scale public health emergency, especially if that emergency could exacerbate preexisting disparities and structural/system-level challenges that would make it more difficult to access necessary resources and services.

During the COVID-19 pandemic, the rate of COVID-19 vaccinations among Black and African American individuals did not reach the same as that of white individuals until March 2022, over one year into the distribution of the vaccine [9]. In anticipation of the next pandemic or another major public health emergency, it is helpful to learn what interventions have been effective in reducing racial disparities in COVID-19 vaccine uptake. This literature review aims to determine “What were the most effective interventions to address COVID-19 vaccine hesitancy among Black and African American individuals in the U.S.?”.

## 2. Materials and Methods

This review followed the Preferred Reporting Items for Systematic Reviews and Meta-Analyses (PRISMA) statement and was registered in PROSPERO (Registration CRD42022371229). This article presents findings on interventions to reduce COVID-19 vaccine hesitancy among Black and African American individuals.

### 2.1. Search Strategy

We searched the Medline, CINAHL, PsycInfo, Cochrane Library, Embase, and Web of Science electronic databases up to 29 December 2022 for relevant peer-reviewed studies published in English and Spanish. The keywords used and the number of articles yielded during the search are listed in Table A1 in the Appendix A. We then used Covidence reference manager software [10] to collect and export the identified studies’ records and eliminate duplicates so they could be more efficiently screened (Veritas Health Innovation 2024). This review followed the same search strategy published in our previous paper [11].

### 2.2. Screening Process and Inclusion/Exclusion Criteria

**Figure 1:**
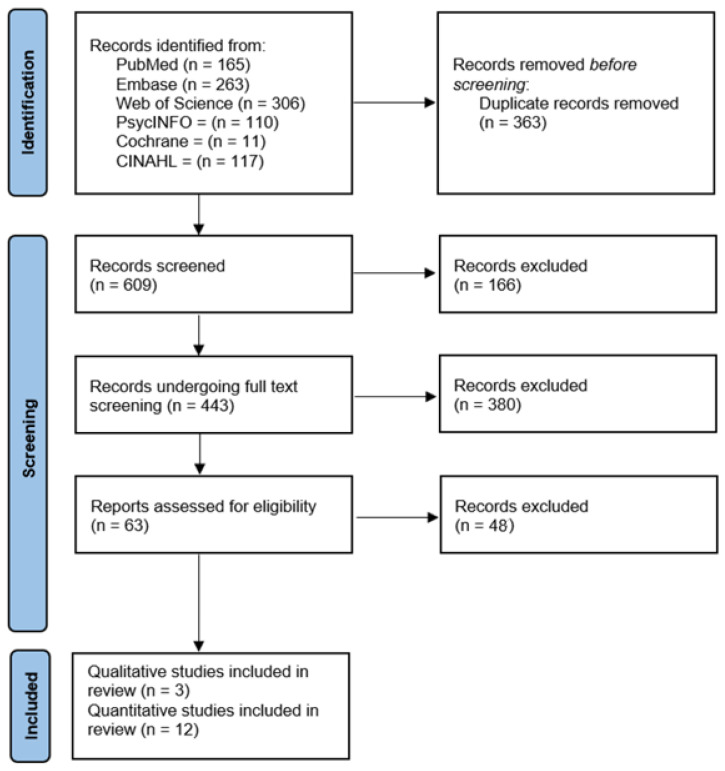
Flowchart of the article screening process.

The screening process consisted of four steps. First, we used Covidence to combine the search results and remove duplicate articles. Then, articles were screened for relevance based on their titles and abstracts. Third, a full-text review was conducted. Information was then extracted based on preestablished criteria. Fourth, the quality of the selected articles was assessed using preidentified tools as described below, and finally the findings were summarized. Figure 1 describes these four steps.

Included studies (a) were based on the U.S. population; (b) described interventions to reduce COVID-19 vaccine hesitancy among Black and African American individuals; (c) were based on primary data collection methods; (d) were peer-reviewed; and (e) were implemented in the field as pilot or full-scale interventions. Excluded studies (a) were not available in full text at our library or through the inter-library services they provide; (b) did not provide information on the interventions intended to change vaccine hesitancy/acceptance; (c) were not reflective of the general population of Black and African American individuals (e.g., a study that only looked at individuals who have a particular rare medical condition); and (d) did not compare the effectiveness of the interventions between Black and African American individuals and other population groups.

### 2.3. Data Extraction

Preestablished criteria were used to develop a standardized form and data extraction Excel document to extract and categorize the selected articles based on (a) study design; (b) sample characteristics (demographics); (c) geographic area; (d) data collection method (e.g., interviews, surveys, or focus groups); (e) type of intervention; (f) outcome measure (how the article defined and measured COVID-19 vaccine hesitancy/acceptance); and (g) results. Two members of the research team (EM and EA) independently reviewed articles and extracted information, meeting to discuss and resolve discrepancies in the categorization process when they occurred. A third team member (ES) would provide the deciding vote if any discrepancies remained after the first two team members met. The extracted data—including the type of intervention, description of the intervention, comparison group, and key findings—is presented in Table 1. The key findings were developed to extract essential practical information for the public health workforce.

## 3. Results

### 3.1. Number of Studies Identified

We identified 12 articles that addressed our literature review question. These 12 articles described the following types of interventions: communication interventions (n = 7), development of partnerships (n = 2), and distribution strategies (n = 3). There was a broad range of study designs describing the use and testing of these interventions: four cross-sectional studies, one longitudinal study, one qualitative study, one retrospective cohort study, one randomized trial, two experimental studies, one observational feasibility study, and one case study. In terms of outcomes, the effectiveness of the intervention was described in terms of vaccination rates (n = 6), vaccine acceptance (n = 2), vaccine hesitancy (n = 3), and vaccine interest and intention to vaccinate (n = 1). It is worth noting that one study used both vaccine acceptance and vaccine hesitancy as the outcome [13]. One additional study was more descriptive in nature and analyzed barriers to vaccination, pilot testing potential interventions. Common overlap among interventions included utilizing technology to deliver messages (n = 9), offering vaccines at established health-care facilities (n = 3), and holding vaccination clinics in non-traditional health facilities, e.g., churches (n = 2).

### 3.2. Type and Impact of the Interventions

The type of intervention described in each of the twelve studies is listed in the Type of Intervention column of Table 1. The impact of these interventions is summarized in the Key Findings column.

### 3.3. Communication Interventions

Interventions on how to best communicate with this segment of the public were described by seven articles, summarized in Table 2. Five of these showed results consistent with one another and two do not. These five articles described the positive impact of four specific interventions: (1) a text message-based outreach program, (2) a mobile application, (3) message content matched to identities, and (4) town halls/information sessions. The remaining two articles provided descriptions of interventions with mixed results on their impact, as shown in Table 2. Below, we provide a description of the interventions and outcomes achieved as described by the studies.

Text message-based outreach program: This intervention described by Ahmed et al. increased uptake of the COVID-19 vaccine among patients by creating a text message-based vaccine outreach program followed with phone calls to schedule an appointment for the vaccination [12]. In this study, they found that Black and African American patients were more likely to schedule a vaccination appointment after receiving a phone call, as an alternative to an email, compared to white patients. They also found that patients who scheduled an appointment were likely to complete their first and second vaccine doses. Unfortunately, in this study results on the actual vaccination uptake were not detailed by race.

Mobile application: Stoner et al. adapted a mobile application for young Black adults called “Tough Talks” that uses choose-your-own adventure (CYOA) narratives to teach users how to disclose their HIV status [18]. They adapted the application by creating narratives related to the COVID-19 vaccination decision-making process and pilot tested it on a small number of users (n = 4). They used feedback from participants and a youth advisory board when pilot testing the application. As a result of the use of this application, Stoner et al. found that relying exclusively on changing social norms, such as stigma from not being vaccinated, was insufficient to increase COVID-19 vaccine acceptance. Other barriers identified by their study include concerns about freedom of choice and vaccine safety.

Message content matched to identities: Dhanani and Franz compared the results of three messaging strategies using a study design with a control group to determine how public health agencies could best increase vaccine acceptance and reduce vaccine hesitancy among Black Americans. The three strategies consisted of (1) providing general information on the vaccination developed by the Centers for Disease Control and Prevention (CDC); (2) presenting the general information developed by CDC and adding a message that stresses the importance of COVID-19 vaccination uptake to reduce racial health disparities; and (3) presenting the general information developed by CDC and adding a message that acknowledges the U.S. medical research industry’s history of abusing Black Americans and describes some of the efforts to promote racial equity during the development and testing of the vaccine [13]. The results of this study show that the most effective communication strategy was the third one acknowledging a past history of unethical research [13].

Gadarian et al.’s cross-sectional study surveyed participants who were recruited through a public opinion research polling firm (YouGov) in order to look at vaccine interest and intention to vaccinate, finding that exposure to messages from same race/ethnicity experts (as opposed to exposure to any vignette) did not significantly improve vaccine interest or intention to vaccinate among racial or ethnic minorities [15].

Reddinger et al. conducted a randomized trial online where they showed to unvaccinated participants messaging about the health risks of COVID-19 to themselves and others and the benefits of getting vaccinated, as well as endorsement from a celebrity (matched to each participant’s identities—Black, Latinx, conservative, religious, or a parent) [17]. Unfortunately, Reddinger et al. did not find any evidence suggesting that adapting communication on COVID-19 vaccination for the real or perceived needs and preferences of broad demographic groups increases the effectiveness of that communication at reducing vaccine hesitancy.

Town halls/information sessions: Feifer et al. reported an increase in the likelihood of Black nursing home employees getting vaccinated compared to white employees after they started having their COVID-19 vaccine information sessions be facilitated by diversity, equity, and inclusion (DEI) representatives [14]. Such sessions were held several times of the day and night and open to employees’ family members. In addition, they featured DEI representatives in their social media campaigns and increased access to multilingual educational materials, leading to increased vaccine acceptance.

Peteet et al. describe the delivery of a 1.5 h-long, dialogue-based webinar on the development of the COVID-19 vaccine and the psychology of fear to Black churchgoing adults, hosted by a community-academic partnership team, to reduce vaccine hesitancy [16]. The intervention resulted in an increase in how many participants reported they would definitely or probably get the COVID-19 vaccine pre-webinar vs. post-webinar. Participants often felt that the most influential aspect of the webinar was the discussion of vaccine facts.

### 3.4. Organizational Partnership Interventions

Interventions focused on how to best utilize organizational partnerships—both preexisting and built as part of a vaccination campaign—to reduce vaccine hesitancy were described by two articles summarized in Table 2. These interventions consisted of developing a three-tiered community-academic partnership model that largely relied on pre-existing relationships and a statewide alliance that leveraged longstanding community partnerships. These interventions were designed to better understand the population’s concerns and the misinformation and racial/ethnic inequities influencing vaccine acceptance. Below, we describe the interventions and outcomes they achieved.

Abdul-Mutakabbir et al. developed a community–academic partnership model to increase the number of COVID-19 vaccines received, resulting in 902 doses being delivered to non-Hispanic Black individuals [19]. This model included holding educational webinars on COVID-19 that were hosted by faith-based organizations and racially concordant faculty, piloting an intervention to provide evidence-informed vaccine education, and holding low-barrier vaccination clinics providing easier access within the community at participating faith leaders’ churches.

Similarly, AuYoung et al. looked at the role of organizational partnerships, i.e., a statewide alliance [20]. Their qualitative study describes the interventions that had been most effective across the 11 academic sites in California that participated in the statewide alliance, named “STOP COVID-19 CA”. The alliance used long-standing community partnerships to more effectively minimize racial inequities in vaccine hesitancy and uptake. AuYoung et al. presented the languages, communication methods, strategies, and trusted messengers that helped these 11 academic sites reduce racial inequities in vaccine hesitancy and uptake. They describe the importance of participating in interviews for Black/African American newspapers and provide recommendations for the development of future coalitions to enhance vaccine uptake such as the value of using trusted community partners to share information or give vaccines.

### 3.5. Distribution Interventions

Interventions focused on reducing barriers to vaccine access through more equitable distribution were described by three articles summarized in Table 2. These interventions included offering COVID-19 vaccines to household members of pediatric patients during scheduled or walk-in well child, ill, and follow-up appointments; setting up accessible vaccination stations (in tandem with holding virtual town halls, establishing a confidential employee hotline, holding department huddles, and providing written educational material); and a retrospective cohort study on the results of offering the vaccine to Medicare patients in a high-touch capitated network. Below, we describe these interventions and outcomes they achieved.

Burkhardt et al. increased COVID-19 vaccine uptake at pediatric primary care practices by offering vaccines to the household members of pediatric patients during scheduled or walk-in well child, sick, and follow-up appointments, administering 2286 doses of the vaccine to 1376 individuals (1270 to patients; 1016 to household members), over half of whom were Black patients [21].

Chan et al. successfully increased COVID-19 vaccine uptake among health-care employees through the rapid deployment of multiple tactics: holding virtual town halls, providing a confidential employee hotline, holding department huddles, offering written educational material, and setting up accessible vaccination stations consisting of walk-up vaccination sites at hospitals and clinics, yielding an increase in complete vaccination rates among Black employees [22].

Lane et al. found through their retrospective cohort study on the relationship between vaccine uptake and access to health care among Medicare patients in a high-touch capitated network spanning 10 states that the odds of being vaccinated increase if someone identifies as Black compared to non-Hispanic white [23]. They collected demographic and clinical data in addition to the date individuals received their COVID-19 vaccine and what type (brand) of vaccine was received from the U.S. census and inpatient and outpatient electronic health records, using logistic regression to determine what factors tend to be associated with higher vaccination rates.

## 4. Discussion

In this manuscript, we describe and summarize the results of interventions that were implemented during the COVID-19 pandemic to enhance COVID-19 vaccine acceptance in Black and African American communities. Public health interventions are effective when they take into account the characteristics of the population and cultural factors that may constitute either barriers or facilitators of the uptake of a program [24]. In this context, race is a cultural determinant of beliefs, behaviors, and both current and historical life experiences that a community may acquire over time. Such determinants may influence the acceptance of a public health program such as a vaccine campaign. In our literature synthesis, we refer to race as a construct defined by its cultural and identity meaning within the U.S. population [25]. Below, we discuss the results focusing on promising practices but also highlighting the paucity of literature and key findings based on our analysis.

We identified 12 studies, 11 of which were focused on the importance of messaging/communication strategies to reduce vaccine hesitancy, making it a very popular type of intervention [12,13,14,15,16,17,18,19,20,22,23]. Two of the twelve studies [15,17] did not find any positive or negative impact of the interventions being implemented on COVID-19 vaccine acceptance. We believe that because of the timing of data collection efforts implemented in these two studies, which occurred later on during the pandemic, the results may reflect the fact that at that point in time the Black and African American individuals had already achieved vaccination rates similar to other groups with little opportunity for improvement [26].

Overall, the majority of the studies we identified show that implementing interventions directed at specific minority groups improves COVID-19 vaccine acceptance without undermining overall vaccine distribution and uptake for other groups. As described by Dhanani and Franz [13], designing their intervention with a major goal of reducing racial inequity in vaccine uptake—particularly for Black and African American individuals—did not undermine the overall allocation of outreach efforts.

Interventions to reduce racial health inequities, especially those that seek to address the underlying causes of COVID-19 vaccine hesitancy among Black and African American individuals, are valuable and a moral necessity because they are an earnest effort to save the lives of individuals who continue to face major institutionalized barriers to health. We can never expect to achieve true health equity without active efforts to reduce existing disparities. During a pandemic, it is important to address vaccine uptake inequities from the start of a vaccination campaign. Lives could have been saved in the Black and African American communities if tailored interventions had been implemented since the development and initial distribution of the vaccine. This literature review identifies some promising practices related to communication, partnerships, and distribution efforts that should be taken into consideration for future pandemics to reduce racial inequities in the uptake of a new vaccine.

## 5. Limitations

We believe that one of the limitations of this study is the fact that we could not conduct a meta-analysis because the studies had such a wide range of outcomes and types of interventions that a quantitative aggregation of results was not possible. In our analysis, we identify themes to present study results in aggregate form and facilitate the uptake of knowledge in an organized manner for the reader. While we were able to identify promising practices, we do not know what intervention is better than others because no study used a comparative approach. We are also aware of the fact that when we use the term “effectiveness,” we are describing study results in a qualitative manner with no possibility of comparisons among implementation efforts. Future research should focus on documenting interventions using implementation science approaches.

## Figures and Tables

**Table 1:** Extracted data.

Citation	Study Design	Sample	Comparison Group	Geographic Area	Setting	Type of Intervention	Outcome Measures	Key Findings *
Communication								
Ahmed et al., 2022 [12]	Longitudinal	30,826 patients at least 65 years old. The majority of patients (24,211/30,826, 78.5%) live in a priority zip code. 19,372 participants were Black or African American	None	Washington, D.C.	Nonprofit health care system	Text message-based intervention using Tavoca platform	(1) Preferred communication method (2) Appointment was scheduled (3) Received the first and second dose	Text messages followed by phone calls were an effective strategy to get African American patients to schedule an appointment to get the COVID-19 vaccination
Dhanani and Franz, 2022 [13]	Experimental	743 participants who resided in the U.S., 244 of whom identified as Black	Control (no messaging)	U.S.	Online	Messaging	Vaccine acceptance and hesitancy	Messaging is an effective strategy to increase vaccine acceptance and reduce vaccine hesitancy among Black Americans
Feifer et al., 2021 [14]	Cross-sectional	Employees at Genesis HealthCare (27,729), 21.0% of whom were Black or African American	Employees from different racial/ethnic groups, especially white employees	U.S.	Long-term care centers	More equitable information sessions, social media campaigns, and access to educational materials	Vaccine acceptance	More equitable information sessions, social media campaigns, and access to educational materials were an effective strategy to increase vaccine acceptance among Black employees
Gadarian et al., 2022 [15]	Cross-sectional	2117 adult respondents, 471 of whom identified as Black	Participants from different racial/ethnic groups, especially white participants, or a control	U.S.	Online	Shared information endorsed by experts	Vaccine interest and intention to vaccinate	Messages from same-race/ethnicity experts was not an effective strategy to improve vaccine interest and intention to vaccinate among racial and ethnic minority groups
Peteet et al., 2022 [16]	Cross-sectional	220 adult Black churchgoers	None	Western U.S.	Virtual	Webinar hosted by an academic- community partnership team	Vaccine hesitancy	A webinar hosted by an academic- community partnership team was an effective strategy to reduce vaccine hesitancy among Black churchgoers
Reddinger et al., 2022 [17]	Randomized trial	3668 unvaccinated respondents, 675 of whom identified as Black or African American	Different racial/ethnic groups, especially those who are religious, conservative, or parents; control	U.S.	Online	Targeted messages	Vaccine hesitancy	Targeted messages matched to broad demographic groups was not an effective strategy to reduce vaccine hesitancy among unvaccinated Black respondents
Stoner et al., 2022 [18]	Experiment	150 Black or African American adults ages 18–29 who are residents of Georgia, Alabama, or North Carolina	None	Georgia, Alabama, and North Carolina	Virtual	Narratives	Development of a digital health intervention	Stoner et al. developed but did not test the effect of a digital health intervention on vaccine hesitancy among Black or African American adults. The intervention was only pilot tested on 4 individuals
Partnerships								
Abdul-Mutakabbir et al., 2022 [19]	Case study	260 adults, 5 of whom identified as Black	None	San Bernardino County, California	Faith-based and community organizations	Partnership between an academic institution and faith and community leaders	Vaccinations received	Partnerships between academia and faith-based and community leaders was an effective strategy to reach non-Hispanic Black communities
AuYoung et al., 2023 [20]	Qualitative	Individuals living in or visiting California at the time of different outreach activities; all ages (percent who identified as Black or African American not stated)	None	California	Virtual and in-person (community health fairs and other community events including community vaccination events; public spaces)	Statewide alliance	Vaccine hesitancy and uptake	A combination of multiple strategies, including communication methods, languages, and trusted messengers were successfully used to reach diverse communities across California. A practical example included participating in interviews for Black/African American newspapers
Distribution								
Burkhardt et al., 2022 [21]	Cross-sectional	Patients in 3 pediatric primary care practices, serving 33,000 children, affiliated with Cincinnati Children’s Hospital Medical Center, 72% of whom identified as Black	Different racial groups	Ohio	3 pediatric primary care practices affiliated with Cincinnati Children’s Hospital Medical Center. Two of the practices were in economically disadvantaged urban settings; the third practice was in the suburbs geographically nearer rural communities	Making vaccines easier to access by offering to administer them during medical appointments	Vaccine uptake	Offering to administer vaccines during medical appointments was an effective strategy to increase vaccine uptake among Black patients and their household members
Chan et al., 2022 [22]	Observational feasibility study	13,942 health-care employees, 566 of whom identified as Black	Different racial/ethnic groups	Washington and Oregon	A health-care organization made up of six hospitals and more than 50 primary care and specialty care clinics spanning two states	Rapid deployment of multiple tactics	Vaccine uptake	The rapid deployment of multiple tactics (virtual town halls, confidential employee hotline, department huddles, written educational material, and accessible vaccination stations) was an effective strategy to increase vaccine uptake among Black employees
Lane et al., 2023 [23]	Retrospective cohort study	93,224 Medicare patients who received care in a high- touch capitated network, 40,201 of whom were non-Hispanic Black	Different racial/ethnic groups	U.S. (Florida, Georgia, Louisiana, Kentucky, Tennessee, Missouri, Virginia, Illinois, Ohio, Pennsylvania, and Texas)	Online	Increased access to health care	Vaccine uptake	Increasing access to health care was an effective strategy to increase vaccine uptake among non-Hispanic Black patients

* These key findings were developed by the authors to extract essential practical information for the public health workforce.

**Table 2:** Description of interventions.

Citation	Name of Intervention	What They Did	Did It Work? (Yes/No)
Communication interventions			
Ahmed et al., 2022 [12]	Text message-based COVID-19 vaccine outreach program	“Developed SMS text messages using the Tavoca platform [to inform] patients of their vaccine eligibility and [allow] them to indicate their interest in scheduling an appointment via a specific method (email or phone) or indicate their lack of interest in the vaccine” (Ahmed et al., 2022)	Yes
Dhanani and Franz, 2022 [13]	Messaging strategies	Used three messaging strategies: general information, general information with a social justice condition, and general information with an ethical oversight condition	Yes
Feifer et al., 2021 [14]	More equitable information sessions, social media campaigns, and access to educational materials	Held COVID-19 vaccine information sessions facilitated by DEI representatives at all times of the day and night that were also open to employees’ family members, featured DEI representatives in their social media campaigns, and improved access to multilingual educational materials	Yes
Gadarian et al., 2022 [15]	Online information from same-race/ethnicity experts	Exposed participants to information on the COVID-19 vaccine that was endorsed by same- or different-race/ethnicity experts vs. any vignette, with a control condition of not seeing a message about vaccination	No
Peteet et al., 2022 [16]	Virtual webinar	Conducted a 1.5 h-long, dialogue-based webinar on the development of the COVID-19 vaccine and the psychology of fear	Yes
Reddinger et al., 2022 [17]	Targeted messages	Showed to unvaccinated participants messaging about the health risks of COVID-19 to themselves and others and the benefits of getting vaccinated as well as endorsement from a celebrity (matched to each participant’s identities—Black, Latinx, conservative, religious, or a parent)	No
Stoner et al., 2022 [18]	Tough Talks—COVID	Adapted the Tough Talks application to apply to COVID-19 vaccination decision-making within social contexts and inspire deeper thought about decisions by creating new choose-your-own adventure narratives	Not stated (only developed and pilot tested)
Partnerships			
Abdul-Mutakabbir et al., 2022 [19]	Community–academic partnership	Developed a community–academic partnership model to increase the number of COVID-19 vaccines received	Yes
AuYoung et al., 2023 [20]	California alliance against COVID-19	Used 11 sites’ strategies, communication methods, languages, and trusted messengers	Yes
Distribution			
Burkhardt et al., 2022 [21]	Offering vaccine to household members	Offered to administer a COVID-19 vaccine to pediatric patients and their household members during the pediatric patient’s appointment	Yes
Chan et al., 2022 [22]	Rapid deployment of multiple tactics	Held virtual town halls, provided a confidential employee hotline, had department huddles, offered written educational material, and set up accessible vaccination stations	Yes
Lane et al., 2023 [23]	Access to health care	Medicare patients received care in a high-touch capitated network, which included providers speaking to them about the benefits of getting a COVID-19 vaccine	Yes

## Data Availability

No new data were created or analyzed in this study. Data sharing is not applicable to this article.

## Appendix A

**Table A1:** Search terms and databases.

Database	Search Terms	Results	Date of Search
PubMed	(“COVID-19 Vaccines”[[M1] Mesh]) AND (“Vaccination Refusal” [Mesh] OR “Vaccin * [M2] Acceptance”) AND (“Race Factors”[Mesh] OR “Racial Groups”[Mesh] OR “black”[Title/Abstract] OR “african american”[Title/Abstract] OR “african-american”[Title/Abstract] OR racia * [Title/Abstract] OR race[Tiab])	*n* = 165	16 November 2022
Embase	(“sars-cov-2 vaccine”/exp OR “coronavirus disease 2019”/exp) AND “vaccine”/exp AND (“vaccine refusal”/exp OR “vaccine hesitancy”/exp OR “vaccine acceptance”/exp) AND (“black”:ti,ab OR “african american”:ti,ab OR “african-american”:ti,ab OR “racia *”:ti,ab OR “race”:ti,ab) AND [embase]/lim	*n* = 263	24 November 2022
Web of Science	(coronavirus OR “corona virus” OR coronavirinae OR coronaviridae OR betacoronavirus OR covid19 OR “covid 19” OR nCoV OR “CoV 2” OR CoV2 OR sarscov2 OR 2019nCoV OR “novel CoV” OR “wuhan virus”) (All Fields) and (vaccin * OR immunizat * OR innoculat * OR booster) (All Fields) and (black OR “african-american” OR “african american”) (All Fields) and (accept * OR hesitan * OR refusal) (All Fields) and Review Article or Article or Early Access (Document Types) and Article or Early Access or Review Article (Document Types) and Article or Early Access or Review Article (Document Types)	*n* = 306	7 December 2022
PsycINFO	Any Field: Coronavirus “vaccin * hesitancy” OR Any Field: “vaccin * refusal” OR Any Field: “vaccin * acceptance” OR Any Field: “vaccin * attitudes” AND Any Field: Black * OR Any Field: race OR Any Field: “Racial and Ethnic Attitudes” AND Any Field: “Covid-19” OR Any Field: “COVID-19”	*n* = 110	20 December 2022
Cochrane	“COVID-19” OR “Coronavirus” OR “Covid-19” OR “coronavirus” OR “corona virus” OR covid19 OR “covid 19” OR “2019nCoV” in All Text AND “Black * [M3] ” OR “african-american” OR “african american” in All Text AND “vaccin * hesitancy” OR “vaccin * refusal” OR “vaccin * acceptance” OR “vaccin * attitudes” in All Text—[M4] (Word variations have been searched)	*n* = 11	23 December 2022
CINAHL	(coronavirus OR “corona virus” OR covid19 OR “covid 19” OR 2019nCoV) (vaccin * OR immunizat * OR innoculat * OR booster) (accept * OR hesitan * OR refusal) (black OR “african-american” OR “african american”)	*n* = 117	29 December 2022

* Boolean operator that indicates that the search result should contain all variations of the root word, stem, or truncation.
